# Disability transitions and life expectancy: a national longitudinal study on social isolation and loneliness in China

**DOI:** 10.1080/16549716.2026.2647315

**Published:** 2026-04-07

**Authors:** Qian Liu, Yuanyuan Li, Fan Yang

**Affiliations:** a School of Public Health, Fudan University, Shanghai, China; b NHC Key Lab of Health Technology Assessment (Fudan University), Fudan University, Shanghai, China

**Keywords:** Social isolation, loneliness, activities of daily living, disability transitions, life expectancy

## Abstract

**Background:**

Functional disability among older adults represents a dynamic process characterized by bidirectional transitions between states rather than a static condition. Social isolation and loneliness have emerged as potentially modifiable determinants of functional outcomes, yet evidence specifically examining their effects on disability state transitions remains limited.

**Objective:**

To examine the independent and interactive associations of social isolation and loneliness with disability state transitions and life expectancy among Chinese adults aged ≥45 years.

**Methods:**

We analyzed data from 20,954 adults aged ≥45 years in the China Health and Retirement Longitudinal Study (2011–2020). Continuous-time multi-state Markov models examined transitions among four states: no disability, mild disability, severe disability, and death. Life expectancy measures were estimated through numerical integration. Four progressively adjusted models evaluated independent and interactive effects.

**Results:**

Social isolation significantly increased risks of transitioning from no disability to mild disability (hazard ratio [HR]=1.19, 95% confidence interval [CI]: 1.06–1.33) and from severe disability to death (HR = 1.33, 95% CI: 1.14–1.54), while reducing recovery likelihood from mild disability (HR = 0.88, 95% CI: 0.78–1.00). Loneliness independently elevated mild disability onset risk (HR = 1.16, 95% CI: 1.06–1.27) and decreased recovery probability (HR = 0.89, 95% CI: 0.81–0.97). Their interaction increased mild-to-severe disability deterioration risk (HR = 1.56, 95% CI: 1.13–2.15). At age 45, social isolation reduced total life expectancy by 3.18 years and loneliness by 2.13 years, primarily through compression of disability-free years.

**Conclusions:**

Social isolation and loneliness synergistically influence disability trajectories by affecting both worsening and recovery transitions, with disproportionate impacts on vulnerable subpopulations.

## PAPER CONTEXT

- **Main findings:** Social isolation and loneliness independently increased the risk of transitioning from functional independence to mild disability while impairing recovery, and their interaction synergistically accelerated progression from mild to severe disability, with social isolation reducing total life expectancy by 3.18 years and loneliness by 2.13 years at age 45, primarily through compression of disability-free years.
- **Added knowledge:** This study advances understanding beyond static disability outcomes by revealing that social disconnection dynamically shapes the entire disability trajectory – influencing not only initial functional decline but also recovery probability and mortality risk among those with severe disability – with disproportionate life expectancy losses among men, rural residents, and individuals with chronic conditions.
- **Global health impact for policy and action:** Routine screening for social isolation and loneliness should be integrated into health assessments for middle-aged and older adults, with comprehensive interventions addressing both structural social networks and subjective emotional connectedness, particularly targeting vulnerable subpopulations facing compounded risks from healthcare access barriers and chronic disease burden.

## Background

Global population aging has fundamentally transformed living arrangements, with solitary living escalating from a marginal phenomenon to epidemic proportions [1]. This demographic shift intersects with mounting evidence that social isolation and loneliness constitute what the World Health Organization (WHO) designates as a ‘silent epidemic,’ with health risks equivalent to smoking 15 cigarettes daily [2]. Social isolation refers to an objective state of limited social networks and infrequent contact, while loneliness represents a subjective, distressing emotional experience arising from perceived relationship deficits [3,4]. Though conceptually distinct, both dimensions particularly affect older populations and have become critical determinants of healthy aging globally.

Contemporary evidence reveals profound health consequences of social disconnection among aging populations. Meta-analytic research demonstrates that stronger social relationships confer a 50% survival advantage, while poor social connections increase coronary heart disease risk by 29% and stroke risk by 32% [3]. Social isolation and loneliness show consistent associations with accelerated cognitive decline, elevated depression prevalence, and increased disability incidence [5]. Beyond mortality and disease endpoints, emerging evidence links social disconnection to functional decline trajectories, with socially isolated older adults exhibiting an accelerated progression through the disability spectrum, from no disability to mild and severe states [6]. Recent multi-cohort analysis of >50,000 older adults internationally found that socially isolated participants face approximately 40% higher risks of progressing to both mild and severe disability within 4 years [7]. A systematic review found that social isolation was associated with 25–30% increased risk of functional decline and disability onset [8]. The English Longitudinal Study of Ageing demonstrated that socially isolated adults showed accelerated progression through disability stages [9]. Similarly, loneliness has been linked to increased incident disability, with effect sizes ranging from 18% to 32% elevated risk [10].

However, this evidence base suffers from three critical limitations that constrain theoretical advancement and intervention design. First, prevailing research conceptualizes disability as a static outcome, neglecting its dynamic transitions across functioning states, including periods of deterioration, stability, and recovery [9,11]. Evidence confirms that functional disability follows complex pathways, with recovery from mild limitations occurring in 20–30% of cases, and that risk factors for disability onset may differ substantially from those influencing recovery or progression [9,12]. A significant gap, therefore, lies in examining how social isolation and loneliness influence the full spectrum of transition intensities between disability states – including onset, recovery, progression, and mortality – within a unified multi-state modeling framework. Understanding whether these social factors primarily accelerate disability onset, hinder recovery, or simultaneously affect multiple transition pathways is essential for determining optimal intervention timing and targeting.

Second, although associations between social isolation/loneliness and functional outcomes are established [7,13], their consequences for the fundamental metrics of population health – total life expectancy (TLE), disability-free life expectancy (DFLE), and life expectancy lived with disability (DLE) – remain unexplored. Social isolation and loneliness may not only trigger the onset of functional limitations but also alter the subsequent trajectory, potentially prolonging the duration of disability and reshaping late-life morbidity patterns. Metrics such as hazard ratios (HRs) are not readily translatable into the healthy life years lost. Quantifying DFLE and DLE offers a powerful, policy-friendly measure of this ‘silent epidemic’s’ impact [14], providing evidence that can directly inform strategies to compress morbidity.

Third, while conceptual distinctions between objective social isolation and subjective loneliness are well established, few studies test whether they exert independent effects on disability state transitions or whether their effects interact [15–17]. Theoretical frameworks suggest that these dimensions operate through partially distinct pathways: objective isolation limits access to instrumental support and health monitoring, while subjective loneliness operates through neuroendocrine stress and behavioral mechanisms [15–17]. However, existing research has not examined whether each dimension’s effect varies across levels of the other throughout disability transitions. This analytical gap constrains intervention design: determining whether to prioritize structural interventions (e.g. increasing social contact) versus psychological interventions (e.g. cognitive-behavioral therapy) requires understanding their independent and interactive effects.

These gaps highlight fundamental uncertainties regarding how social isolation and loneliness shape disability state transitions, DFLE, and life expectancy with disability. China offers a critical setting to address these questions, possessing the world’s largest aging population, which is experiencing rapid social transformations that potentiate risks of objective isolation and subjective loneliness [18]. This study employs a multi-state transition model to analyze how social isolation and loneliness govern transitions across disability states among Chinese adults aged 45 years and older. We further quantify their differential impacts on TLE, DFLE, and years lived with disability, thereby providing an evidence base for interventions tailored to specific transition stages.

## Methods

### Study design and population

We utilized data from five waves (2011–2020) of the China Health and Retirement Longitudinal Study (CHARLS), a nationally representative survey conducted by Peking University. This study employed a multistage stratified probability proportionate to size (PPS) sampling strategy, ultimately covering 28 provinces, 150 county-level units, and 450 village-level units. While detailed survey procedures and response rates have been reported elsewhere [19,20], comprehensive methodological details for CHARLS are documented in Methods A.1 in the Supplement. Our analysis was conducted following the Strengthening the Reporting of Observational Studies in Epidemiology guidelines. We restricted the analytic sample to participants aged 45 and older from the CHARLS study between 2011 (Wave 1) and 2020 (Wave 5). We excluded 260 participants due to missing disability state information at any assessment wave, 2393 individuals with missing baseline social isolation, loneliness, and covariate data, and 1821 participants with only single-wave disability data available. This methodology aligns with established multi-state modeling practices requiring participants to have multiple observed disability states during follow-up periods to enable transition analyses [21]. After these sequential exclusions, 20,954 participants were included in the final analysis (Figure A.1).

### Assessment procedure

#### Assessment of exposures

In the CHARLS cohort, social isolation and loneliness were assessed through self-reported questionnaires. For social isolation assessment, participants received 1 point for each of the following: living alone, being unmarried (including separated, divorced, widowed, or never married), having less than weekly contact with children (via telephone, face-to-face, or email), and not participating in any social activities in the past month (including interacting with friends, playing chess or cards, or participating in sports, social, or other clubs). The social isolation index ranged from 0 to 4, with higher scores indicating greater social isolation. Participants scoring ≥2 were classified as socially isolated [22,23].

For loneliness assessment, a single item from the Center for Epidemiologic Studies Depression Scale (CES-D) was used: ‘How often did you feel lonely in the past week?’ Participants were classified as lonely if they reported feeling lonely occasionally (1–2 days per week), frequently (3–4 days per week), or most of the time (5–7 days per week). Those reporting feeling lonely rarely or never (less than 1 day per week) were classified as not lonely [13,22]. While single-item assessment has inherent limitations, this approach aligns with widely used measures in large-scale epidemiological studies and demonstrates satisfactory concurrent validity with multi-item loneliness scales in older populations [7,13]. This measure effectively captures the core affective component of loneliness and has been validated against the UCLA Loneliness Scale across multiple cohorts [15]. However, it is important to note that a single-item measure may not fully capture the multidimensional nature of loneliness, including emotional and social subtypes, which have been shown to exert differential effects on health outcomes in older adults [24,25]. Consequently, the associations observed in this study likely reflect the global subjective experience of loneliness rather than its specific dimensions, and the magnitude of certain pathway-specific effects may be attenuated due to measurement imprecision.

A related methodological concern pertains to the temporal scope of this measure. Although the item references the past week, loneliness exhibits substantial temporal stability in older populations (test–retest reliability: 0.73–0.85 over months to years [26]), reflecting an enduring psychological state rather than a transient mood. The past-week timeframe captures recent affective experiences that may better predict health outcomes than abstract lifetime assessments. Our repeated-measures design (five waves over 9 years) further mitigates concerns about momentary fluctuations, though this approach may be more sensitive to recent changes in social circumstances than measures with longer reference periods. Nevertheless, the use of five waves of data spanning 9 years allows for the examination of loneliness as a time-varying exposure, such that any wave-specific measurement error is unlikely to systematically bias the longitudinal associations observed.

#### Functional disability assessment

Based on prior research, functional disability was assessed using standardized measures of basic and instrumental activities of daily living (ADL/IADL). ADL comprised six self-care tasks: dressing, bathing, eating, getting in and out of bed, toileting, and controlling urination and defecation. IADL covered six higher-order functional domains: making phone calls, preparing hot meals, shopping for groceries, performing household chores, taking medications, and managing finances. For each task, participants reported whether they could perform the task independently or required assistance. For multi-state Markov modeling, we defined four distinct health states: (a) No disability; (b) Mild disability (IADL limitations only); (c) Severe disability (≥1 ADL limitation, with or without IADL limitations) [13], and (d) Death. This schema captures the severity spectrum of disability, allowing direct transitions between all living states to model heterogeneous pathways of onset, progression, and recovery [27].

#### Covariate assessment

Covariates were selected based on their established associations with social isolation, loneliness, and functional disability transitions. Sociodemographic variables included gender, age (categorized as 45–54, 55–64, 65–74, and ≥75 years), educational attainment (less than secondary school vs. secondary school or above), and residential location (urban vs. rural). Behavioral factors encompassed smoking history and alcohol consumption history. Health status incorporated chronic disease presence and depressive symptoms assessed using the CESD-9, which denotes the modified Center for Epidemiological Studies Depression Scale with the loneliness item excluded. Additional details are provided in Table A.1 in the Supplement.

### Statistical methodology

Continuous-time multi-state Markov models were applied to examine transitions between disability states using the msm package in R [21]. Four states were specified: no disability, mild disability, severe disability, and death. Models assumed unobserved exact transition times, potential multiple transitions between observations, and precisely recorded death dates [28]. Transition pathways were specified based on observed patterns, excluding rare transitions to maintain model stability [29]. We fitted four models progressively incorporating social isolation (Model 1), loneliness (Model 2), both dimensions (Model 3), and their interaction (Model 4), all adjusted for covariates. The framework generated HRs with 95% confidence intervals (CIs) representing instantaneous transition rates. Transition probabilities were estimated as competing event probabilities [21]. Mean sojourn times quantified average duration in each state [30]. Life expectancy measures were computed using the elect package in R [21], fitting multinomial regression models to estimate total and marginal life expectancy from multi-state parameters [31]. TLE represented the expected remaining lifespan. Marginal life expectancy calculated expected years in each living state (no disability, mild disability, severe disability). Estimates were generated at 5-year intervals from ages 50 to 90 years. Stratified analyses by gender, residence, and chronic disease status evaluated differential impacts of social isolation and loneliness across population subgroups. Multiple sensitivity analyses were conducted to evaluate the robustness of our findings. First, we restricted the sample to participants aged 50 years and older. Second, we implemented alternative disability definitions based solely on ADL limitations, excluding IADL criteria. Third, we fitted models using participants aged 50 and older with time-varying covariates updated at each wave, compared to our primary analysis using baseline covariates among those aged 45 and older, to evaluate potential time-dependent confounding. Fourth, we applied simplified three-state models (no disability, any disability, death) to examine consistency across different state specifications. These sensitivity analyses confirmed the stability of our results under varying methodological assumptions, providing confidence in the validity and robustness of the observed associations.

All statistical analyses were performed using R software, with significance levels set at *p* < 0.05 for hypothesis testing.

## Results

### Participant characteristics

Table 1 shows that this study included 20,954 Chinese adults aged 45 years and above from the CHARLS. The sample was relatively young, with 76.6% aged 45–64 years and 23.3% aged 65 years or older. Gender distribution was approximately balanced (51.6% female), while geographic distribution showed rural predominance (59.4%), and educational attainment was predominantly low, with 68.2% having less than secondary school education. Health and lifestyle characteristics indicated that 40.4% reported a history of smoking and 42.6% a history of alcohol consumption. Chronic disease prevalence was substantial at 42.2%, and half of the participants (50.3%) exhibited depressive symptoms based on the modified CESD-9 scale (scores ≥9). Social characteristics demonstrated strong familial and community connections: the vast majority lived with others (95.4%), were married or partnered (89.5%), and maintained at least weekly contact with their children (92.1%), while 48.9% participated in social activities. Regarding the primary exposure variables, social isolation prevalence was 11.6%, whereas loneliness was more common at 27.9%, indicating a notable distinction between objective social disconnection and subjective feelings of loneliness in this older Chinese population.

**Table 1.** Baseline characteristics of the participants (*N* = 20,954).

Variable	Category	Number (constituent ratio [%])
Age group	45–54	9048 (43.2%)
	55–64	7007 (33.4%)
	65–74	3527 (16.8%)
	≥75	1372 (6.5%)
Gender	Female	10,810 (51.6%)
	Male	10,144 (48.4%)
Residence	Rural	12,443 (59.4%)
	Urban	8511 (40.6%)
Education level	Less than secondary school	14,283 (68.2%)
	secondary or above	6671 (31.8%)
Smoke	No	12,494 (59.6%)
	Yes	8460 (40.4%)
Drink	No	12,020 (57.4%)
	Yes	8934 (42.6%)
Chronic disease	No	12,119 (57.8%)
	Yes	8835 (42.2%)
CESD-9	No	10,410 (49.7%)
	Yes	10,544 (50.3%)
Loneliness	No	15,114 (72.1%)
	Yes	5840 (27.9%)
Social isolation	No	18,522 (88.4%)
	Yes	2432 (11.6%)
Living alone	No	19,984 (95.4%)
	Yes	970 (4.6%)
Marital status	No	2194 (10.5%)
	Yes	18,760 (89.5%)
Less than weekly contact with children	No	1648 (7.9%)
	Yes	19,306 (92.1%)
Participate in social activities	No	10,700 (51.1%)
	Yes	10,254 (48.9%)

Note: CESD-9 refers to the Center for Epidemiological Studies Depression Scale excluding the loneliness item (range 0–27); scores ≥9 indicate depressive symptoms.

### Transition probabilities between states

One-year transition probabilities revealed distinct disability dynamics across states (Table 2). Functional independence demonstrated strong stability, with 91.49% of individuals without disability remaining in that state over 1 year. Among those with mild disability, recovery to functional independence occurred in 24.24% of cases, while 63.39% remained in the same disability state and 10.39% progressed to severe disability. Individuals with severe disability showed limited recovery, with only 8.03% returning to functional independence and 15.00% improving to mild disability, while 63.78% remained in severe disability. Mortality risk varied substantially by disability status: the one-year mortality probability was minimal among those without disability (0.21%), low among those with mild disability (1.98%), but markedly elevated among those with severe disability (13.18%), representing a 63-fold increase compared to the functionally independent group.

**Table 2.** One-year transition probabilities between disability states (unadjusted).

	No disability	Mild disability	Severe disability	Death
No disability	**0.9149**	0.0653	0.0177	0.0021
Mild disability	0.2424	**0.6339**	0.1039	0.0198
Severe disability	0.0803	0.1500	**0.6378**	0.1318

Note: Values represent the probability (%) of transitioning from the initial state (rows) to the outcome state (columns) over a one-year period. Rows sum to 100%. Diagonal values (in bold) represent the probability of remaining in the same state.

### Mean sojourn times

Mean sojourn times indicated that disability states exhibited similar durations across the categories (Table 3). The mean duration of functional independence was 9.85 years (95% CI: 9.60–10.12), substantially longer than the durations of disability states. In contrast, each episode of mild disability lasted an average of 2.04 years (95% CI: 1.97–2.11), while severe disability episodes persisted for 2.13 years (95% CI: 2.03–2.23), showing comparable durations between the two disability states. These findings suggest that once disability develops, the duration of impairment remains relatively consistent regardless of the severity level.

**Table 3.** Mean sojourn times in each disability state (unadjusted).

	Estimates	SE	L	U
State 1	9.85	0.13	9.60	10.12
State 2	2.04	0.04	1.97	2.11
State 3	2.13	0.05	2.03	2.23

Note: State 1 = no disability; State 2 = mild disability; State 3 = severe disability; State 4 = death. SE = standard error; L = lower limit of 95% confidence interval; U = upper limit of 95% confidence interval.

### Associations between social isolation, loneliness, and disability transitions

Table 4 presents the associations between social isolation, loneliness, and disability state transitions across four progressive models. In Model 1, social isolation independently increased the risk of transitioning from no disability to mild disability (HR = 1.23, 95% CI: 1.10–1.37, *p* < 0.001) and from severe disability to death (HR = 1.32, 95% CI: 1.14–1.53, *p* < 0.001), while decreasing the likelihood of recovery from mild disability to no disability (HR = 0.87, 95% CI: 0.77–0.98, *p* < 0.05). Model 2 showed that loneliness similarly increased the risk of progression from no disability to mild disability (HR = 1.19, 95% CI: 1.09–1.30, *p* < 0.001) and reduced the likelihood of recovery from mild to no disability (HR = 0.89, 95% CI: 0.81–0.97, *p* < 0.05).

**Table 4.** HRs for state transitions associated with social isolation and loneliness.

	Model 1	Model 2	Model 3		Model 4		
	Social isolation	Loneliness	Social isolation	Loneliness	Social isolation*loneliness	Social isolation	Loneliness
	HR (95%CI)	HR (95%CI)	HR (95%CI)	HR (95%CI)	HR (95%CI)	HR (95%CI)	HR (95%CI)
State 1–State 2	1.23*** (1.10,1.37)	1.19*** (1.09,1.30)	1.19** (1.06,1.33)	1.16** (1.06,1.27)	0.85 (0.69,1.05)	1.27*** (1.10,1.47)	1.20*** (1.09,1.33)
State 1–State 3	1.26 (0.92,1.73)	1.23 (0.97,1.55)	1.19 (0.86,1.64)	1.20 (0.95,1.53)	0.98 (0.54,1.78)	1.22 (0.80,1.86)	1.22 (0.95,1.56)
State 2–State 1	0.87* (0.77,0.98)	0.89* (0.81,0.97)	0.88* (0.78,1.00)	0.89* (0.81,0.97)	1.00 (0.79,1.27)	0.87 (0.74,1.03)	0.89* (0.81,0.99)
State 2–State 3	1.02 (0.87,1.20)	1.08 (0.94,1.25)	1.01 (0.86,1.19)	1.08 (0.93,1.25)	1.56** (1.13,2.15)	0.80 (0.63,1.01)	0.97 (0.82,1.15)
State 2–State 4	0.90 (0.51,1.58)	0.97 (0.66,1.43)	0.90 (0.50,1.63)	1.01 (0.66,1.54)	0.60 (0.19,1.88)	1.16 (0.63,2.14)	1.09 (0.70,1.69)
State 3–State 1	1.38 (0.86,2.22)	0.68 (0.44,1.05)	1.38 (0.86,2.24)	0.72 (0.47,1.10)	1.90 (0.77,4.68)	1.12 (0.60,2.07)	0.58* (0.34,0.99)
State 3–State 2	0.87 (0.68,1.11)	1.01 (0.84,1.21)	0.88 (0.69,1.13)	1.01 (0.84,1.21)	1.47 (0.91,2.36)	0.69 (0.47,1.01)	0.96 (0.79,1.17)
State 3–State 4	1.32*** (1.14,1.53)	0.99 (0.86,1.15)	1.33*** (1.14,1.54)	0.95 (0.82,1.10)	1.10 (0.82,1.47)	1.26* (1.03,1.55)	0.92 (0.77,1.10)

Note: Model 1: social isolation as predictor; Model 2: loneliness as predictor; Model 3: both social isolation and loneliness as predictors; Model 4: model 3 + isolation*loneliness interaction. HR: hazard ratio; CI: Confidence Interval. State 1 = no disability; State 2 = mild disability; State 3 = severe disability; State 4 = death; * indicates 5% significant ratio. *p < 0.05, **p < 0.01, ***p < 0.001. All models were adjusted for age, gender, residence, education level, chronic diseases, healthy behaviors (drink and smoke), and depression.

When both factors were included in Model 3, social isolation remained significantly associated with mild disability onset (HR = 1.19, 95% CI: 1.06–1.33, *p* < 0.01), impaired recovery from mild disability (HR = 0.88, 95% CI: 0.78–1.00, *p* < 0.05), and mortality from severe disability (HR = 1.33, 95% CI: 1.14–1.54, *p* < 0.001). Loneliness maintained independent associations with mild disability onset (HR = 1.16, 95% CI: 1.06–1.27, *p* < 0.01) and reduced recovery from mild disability (HR = 0.89, 95% CI: 0.81–0.97, *p* < 0.05).

Model 4, which incorporated an interaction term, revealed a synergistic effect: the combined presence of social isolation and loneliness significantly increased the risk of progression from mild to severe disability (HR = 1.56, 95% CI: 1.13–2.15, *p* < 0.01), indicating that their interaction accelerates disability deterioration. Additionally, in this model, loneliness was associated with a reduced likelihood of recovery from severe disability to no disability (HR = 0.58, 95% CI: 0.34–0.99, *p* < 0.05). An older adult with mild disability who is both socially isolated and lonely faces a substantially elevated risk of progressing to severe disability compared to someone experiencing only one of these conditions or neither. Specifically, the interaction term in Model 4 yielded an HR of 1.56 (95% CI: 1.13–2.15, interaction *p* < 0.01), indicating that the co-occurrence of social isolation and loneliness is associated with a 56% higher risk of progression from mild to severe disability beyond what would be expected from either condition alone. It should be noted that social isolation here refers to the objective lack of social contacts or network connections, whereas loneliness reflects the individual’s subjective self-reported feeling of being lonely.

To further examine whether loneliness in the absence of social isolation confers distinct health effects, we constructed a four-category joint exposure variable (neither isolated nor lonely [reference]; isolated only; lonely only; both isolated and lonely) and fitted it within the multi-state Markov model. Full results are presented in Table A.2. Individuals who were lonely only showed elevated risks of disability onset (no disability to mild disability: HR = 1.22, 95% CI: 1.06–1.40, *p* < 0.01; no disability to severe disability: HR = 1.39, 95% CI: 1.23–1.57, *p* < 0.001) without significant effects on recovery or mortality transitions, suggesting that subjective loneliness primarily accelerates the initiation of functional decline. Isolated only individuals showed impaired recovery from mild disability (HR = 0.77, 95% CI: 0.61–0.98, *p* < 0.05) and elevated mortality risk from severe disability (HR = 1.26, 95% CI: 1.07–1.47, *p* < 0.01), reflecting a pattern consistent with structural resource deprivation. Those experiencing both conditions demonstrated worsened disability progression (HR = 1.40, 95% CI: 1.10–1.77, *p* < 0.01) and elevated mortality from severe disability (HR = 1.21, 95% CI: 1.04–1.41, *p* < 0.05), indicating a compounding burden. All models were adjusted for age, gender, residence, education, chronic diseases, health behaviors, and depression.

### Associations between social isolation, loneliness, and life expectancy

Table 5 presents the disability-specific life expectancy disparities associated with social isolation and loneliness.

**Table 5.** Differences in life expectancy by age and disability status associated with social isolation and loneliness.

	Age	No disability	Mild disability	Severe disability	Total
Social isolation	45	−2.73***	−0.12	−0.33*	−3.18***
	50	−2.48***	−0.13	−0.33*	−2.94***
	55	−2.20***	−0.14	−0.34*	−2.68***
	60	−1.91***	−0.18	−0.35*	−2.44***
	65	−1.57***	−0.18	−0.37*	−2.13***
	70	−1.26***	−0.17	−0.35*	−1.79***
	75	−0.97***	−0.15	−0.35*	−1.47***
	80	−0.71***	−0.12	−0.32*	−1.15***
	85	−0.51***	−0.07	−0.25	−0.84**
	90	−0.36**	−0.01	−0.17	−0.54**
Loneliness	45	−2.72***	0.29	0.30	−2.13***
	50	−2.53***	0.23	0.31	−1.99***
	55	−2.28***	0.16	0.28	−1.84***
	60	−2.00***	0.05	0.26	−1.69***
	65	−1.69***	−0.01	0.23	−1.47***
	70	−1.36***	−0.06	0.21	−1.20***
	75	−1.05***	−0.12	0.18	−1.00**
	80	−0.76***	−0.13	0.16	−0.74*
	85	−0.55***	−0.12	0.15	−0.52
	90	−0.38***	−0.10	0.15	−0.32

Note: *p < 0.05, **p < 0.01, ***p < 0.001.

Both exposures were associated with significant reductions in TLE, with the magnitude of the reduction diminishing progressively at older ages. At age 45, social isolation was associated with a reduction in TLE of 3.18 years (*p* < 0.001), while loneliness was associated with a reduction of 2.13 years (*p* < 0.001), indicating a greater absolute loss for social isolation.

However, the composition of these losses differed substantially. Both primarily reduced disability-free life years (social isolation: −2.73 years; loneliness: −2.72 years at age 45, both *p* < 0.001). Critically, social isolation was additionally associated with shorter life expectancy with severe disability (−0.33 years at age 45, *p* < 0.05), whereas loneliness showed no significant association with either mild or severe disability life expectancy.

This divergent pattern reveals distinct consequences. While social isolation is associated with reductions in both healthy years and time with severe disability, loneliness is linked to reduced TLE almost exclusively through loss of healthy years. Social isolation reduces both disability-free years (−2.73 years) and years lived with severe disability (−0.33 years), meaning it shortens survival across both healthy and disabled states. Loneliness, by contrast, reduces TLE almost entirely through the loss of disability-free years (−2.72 years), with no significant reduction in years lived with disability. In other words, loneliness compresses the healthy portion of the remaining life while leaving the disabled portion largely intact.

### Stratified analysis by gender, chronic disease status, and residence

Stratified analyses revealed distinct patterns in the association between social isolation and disability-specific life expectancy (Figures A.2–A.7). A marked gender disparity was observed, with males exhibiting significantly greater reductions in both TLE (e.g. −3.40 vs. −2.50 years at age 45) and DFLE (e.g. −3.04 vs. −2.26 years at age 45) compared to females. When stratified by chronic disease status, both groups showed significant reductions in total and DFLE; however, individuals with chronic diseases additionally exhibited a significant reduction in years lived with severe disability throughout the age range of 55–80 years (e.g. −0.40 years at age 55 and −0.36 years at age 80). Regarding residence, both urban and rural residents showed significant reductions in TLE and DFLE across all age groups. The key distinction emerged in years lived with severe disability: rural residents demonstrated a significant reduction from age 50 to 85 (e.g. −0.35 years at age 50 and −0.28 years at age 85), a pattern largely absent in urban counterparts.

Stratified analyses for loneliness revealed its consistent association with reduced DFLE across all demographic subgroups (Figures A.8–A.13), with no substantial variations by gender, chronic disease status, or residence. A distinct gender disparity was observed in TLE: males showed slightly larger reductions than females (e.g. −2.17 vs. −1.83 years at age 45), and this significant association persisted longer into older ages for males (up to age 85) compared to females (up to age 75). Across all stratifications, loneliness demonstrated no significant association with life expectancy in mild or severe disability states.

### Sensitivity analyses

Sensitivity analyses confirmed the robustness of the primary findings regarding the associations of social isolation and loneliness with disability transitions. Across all model specifications – including raising the baseline age to 50 years, using an ADL-only disability definition, and employing wave-complete data – the core associations remained largely consistent (Table A.3). Specifically, social isolation maintained significant associations with increased risks of transitioning from no disability to mild disability and from severe disability to death in most sensitivity models. Loneliness consistently demonstrated associations with mild disability onset and impaired recovery from mild disability. The simplified three-state model further validated these patterns, showing significant associations between social isolation/loneliness and increased risks of transitioning from no disability to disability, while loneliness was significantly associated with impaired recovery from disability to functional independence (Table A.4).

## Discussion

This study shows that social isolation and loneliness are associated with differentiated patterns of disability transitions, not only increasing the risk of worsening from a non-disabled state to mild disability but also influencing the subsequent risk of either further deterioration or recovery. Rather than reflecting a general decline in functioning, these exposures are linked to a higher risk of transitioning from no disability into mild disability and a reduced likelihood of returning from mild disability to a non-disabled state. Social isolation was further associated with elevated mortality risk among individuals already in severe disability, contributing to reductions in both TLE and life expectancy within the disabled state. Loneliness also exhibited independent associations with both worsening and impaired recovery transitions, although its effects were concentrated primarily in DFLE. These findings suggest that deficits in social relationships are embedded in the process of disability transitions itself, shaping the pattern of deterioration and recovery risks rather than merely the presence or absence of disability.

Our findings align with and extend previous research in several important ways. The independent effects of social isolation (HR = 1.19) and loneliness (HR = 1.22) on disability onset are consistent with meta-analytic estimates [32,33]. Crucially, our study demonstrates that these factors also significantly impair recovery from disability (12% and 15% reductions in recovery likelihood, respectively) – an aspect rarely examined in prior work. A Health and Retirement Study reporting that social support increased disability recovery rates by approximately 16% [34] provides convergent evidence from an inverse perspective. Furthermore, the significant interaction between social isolation and loneliness on progression from mild to severe disability (HR = 1.34) represents a novel contribution, as few prior studies have tested for such interactive effects despite their theoretical plausibility [35].

Our life expectancy estimates translate HR findings into interpretable public health metrics. Notably, social isolation and loneliness exhibited contrasting patterns in how they erode life expectancy. Social isolation reduced both disability-free years (−2.73 years) and years with severe disability (−0.33 years), indicating a broadly distributed impact across survival with and without disability. In contrast, loneliness reduced TLE by 2.13 years almost entirely through the loss of disability-free years (−2.72 years), with minimal impact on years lived with disability. This pattern is consistent with previous work suggesting that loneliness operates predominantly through disability pathways rather than direct mortality pathways [36]. Taken together, these results indicate that loneliness primarily compresses the healthy period of life while leaving the duration of disability largely intact, whereas social isolation shortens both healthy survival and survival within the severe disability state – an important distinction with implications for targeted intervention design.

Divergent life expectancy patterns observed for social isolation and loneliness can be understood through their distinct underlying mechanisms. Social isolation, as an objective structural deficit in social relationships, influences disability transitions through at least four interconnected pathways. First, the lack of social contact restricts opportunities for engaging in externally oriented daily activities, which inherently require interaction with others [37]. This deprivation of external stimuli may lead to reduced cognitive and executive function, thereby increasing the risk of worsening from a non-disabled state to mild disability and decreasing the likelihood of recovery once disability has occurred. Second, the depletion of social networks limits instrumental and informational support when early functional decline emerges [38], reducing the chances of obtaining timely medical or rehabilitative assistance and thus hindering recovery. Third, social isolation is linked to elevated chronic inflammation and decreased physiological resilience [32,39], which compromises the body’s ability to maintain or restore function, contributing to higher risks of deterioration and lower recovery probability. Finally, the absence of organized social participation – such as in clubs or religious activities – has been associated with functional disability [37]; these activities provide cognitive and emotional stimulation that may support transitions toward recovery, and their absence may instead promote deterioration. These same mechanisms also explain why social isolation reduces life expectancy in the severe disability state. Individuals with severe disability depend heavily on external support for daily living, and the absence of close relationship networks prevents timely access to medical assistance when acute symptoms arise, accelerating the transition from disability to death [38]. The unhealthy behaviors and chronic inflammation associated with social isolation [32,39] further exacerbate health risks among the severely disabled, shortening survival duration in this state.

Loneliness, as a subjective emotional experience, influences the risks of deterioration and recovery transitions through both psychological and behavioral pathways. It represents a chronic stressor that elevates cortisol levels and disrupts biological homeostasis [40], thereby weakening physiological resilience and increasing the risk of worsening between disability states. In addition, loneliness is associated with health-risk behaviors such as smoking, alcohol misuse, and physical inactivity [41], as well as with psychological distress including depression and anxiety [42], which further impair recovery from mild disability. Some studies have reported that the relationship between loneliness and physical functioning becomes non-significant after comprehensive adjustment for health and social factors [13], suggesting that loneliness may partially arise as a consequence of underlying health problems and that its impact may be overestimated when objective social relationships are not considered [43]. Nevertheless, in the present study, loneliness remained independently associated with both deterioration and recovery transitions after controlling for social isolation, indicating that its effects extend beyond objective social connections. Prior research has suggested that psychological stress and health-risk behaviors mediate the link between loneliness and adverse health outcomes [44], implying that loneliness influences the risk structure of disability transitions through emotional and behavioral pathways distinct from those of social isolation. These predominantly non-mortality pathways are consistent with the observed life expectancy pattern, in which loneliness reduced disability-free years substantially while leaving survival duration within the disability state largely unaffected.

A notable gender difference emerged: men experiencing social isolation showed greater reductions in both disability-free and TLE than women. Men rely predominantly on spousal relationships to maintain functional health, whereas women tend to develop broader networks that include spouses, children, and community members [45,46]. For men, loss of marital ties represents depletion of their primary social support, whereas women retain emotional connections through alternative relationship channels even when meeting social isolation criteria. Widowed men, in particular, often struggle to maintain social connections that were previously sustained through their spouses [47]. Biologically, social isolation triggers stronger inflammatory responses among men, characterized by higher fibrinogen levels and greater cumulative inflammatory burden [48], which may contribute to accelerated physiological decline and explain the greater magnitude of reduction in disability-free and TLE compared with women.

The association between social isolation and life expectancy in the severe disability state was pronounced among rural residents and individuals with chronic diseases but largely absent in urban or non-chronically ill populations. In rural China, inadequate healthcare infrastructure – only about 61% of residents can reach medical services within 1 km compared with 82% in urban areas – combined with social isolation, leaves ADL-disabled individuals without both informal caregiving support and timely access to professional medical services [49,50]. This dual deprivation accelerates the transition from severe disability to death, significantly reducing life expectancy in the severe disability state. Among individuals with chronic diseases, the conditions themselves elevate the mortality risk and accelerate functional deterioration. Social isolation compounds these challenges by hindering disease management and reducing treatment adherence [51,52], causing individuals with severe disability to transition more rapidly toward death and experience shorter survival duration in this state.

These findings carry important implications for public health and clinical practice in aging societies. Routine health evaluations should include screening for social isolation and loneliness, particularly among men, rural residents, and individuals with chronic diseases who are more susceptible to adverse disability trajectories. Interventions targeting social isolation – such as group-based activities, social skills training, and community support programs [53,54] – can help preserve functional ability, delay disability onset, and extend DFLE. For those already disabled, strengthening social networks may slow the progression to death and prolong life expectancy within the disability state. Given the synergistic effects observed between social isolation and loneliness, particularly in the progression from mild to severe disability, comprehensive strategies that address both structural and emotional aspects of social connection are essential. Social prescribing in primary care and telehealth services in rural areas may together extend disability-free years and reduce post-disability mortality [55,56], requiring coordinated actions across policy, healthcare, and family levels.

While our findings are derived from the Chinese context, several features support their broader relevance. The prevalence rates of social isolation (10–15%) and loneliness (20–30%) observed in our study are comparable to those reported across European, North American, and other Asian populations [54,57], and the dynamic patterns of disability transitions and recovery potential observed here align with those documented in international cohorts [58,59]. Critically, the finding that co-occurring social isolation and loneliness significantly accelerate progression to severe disability – and that loneliness independently impairs recovery – points to a modifiable dual-risk profile that is not unique to China. As populations age globally, policymakers and public health practitioners in other countries may similarly benefit from screening for both social isolation and loneliness as distinct yet compounding risk factors, and from designing integrated interventions that address both objective social disconnection and subjective feelings of loneliness simultaneously, rather than treating them as interchangeable constructs.

Several limitations warrant careful interpretation of results. First, while our single-item loneliness measure offers practical advantages for large-scale surveillance, it cannot capture the multidimensional nature of loneliness (e.g. emotional versus social loneliness) or distinguish between transient situational loneliness and chronic loneliness patterns. The binary classification may also obscure dose–response relationships, though this approach enhances clinical interpretability and aligns with prior research establishing clinically meaningful thresholds. Second, the study relied on self-reported data, raising potential concerns regarding common method bias. Disability states were determined based on self-reported ADL and IADL difficulties, which may be influenced by reporting bias and subjective perception. Third, certain aspects of social relationships, such as contact with grandchildren and community members, were not incorporated into the analysis, potentially overlooking social connection dimensions that hold particular significance within collectivistic cultural contexts. Fourth, the research sample comprised Chinese middle-aged and older adults, and generalizability of findings may be limited by cultural specificity, particularly regarding family structure, intergenerational support, and social norms that differ fundamentally from Western societies. Future research should employ multi-dimensional loneliness measures, incorporate objective assessments alongside self-reports, examine broader social relationship networks, and test whether similar patterns emerge across diverse cultural settings.

## Conclusion

By tracking bidirectional transitions across multiple disability states, this study reveals a critical insight often obscured in cross-sectional research: social disconnection operates as a dynamic force throughout the entire disability trajectory – not merely predicting initial functional decline but substantially influencing whether individuals recover independence or deteriorate toward premature mortality. The overlapping influences of social isolation and loneliness on worsening and recovery transitions, combined with their synergistic effect in promoting mild disability deterioration, underscore that deficits in objective social connections and subjective emotional experiences create compounded vulnerability requiring comprehensive intervention. Importantly, these associations operate inequitably – men, rural residents, and individuals with chronic diseases experience disproportionately greater losses in life expectancy under social isolation, revealing how social disconnection amplifies existing vulnerabilities rooted in healthcare access barriers and chronic disease burden. This reframes disability prevention in aging societies: effective strategies must shift from merely delaying functional loss to actively strengthening social relationships that enable functional restoration and prevent the transition from severe disability to premature death among those most at risk.

## Supplementary Material

Supplementary material_order.docx

## Data Availability

The data that support the findings of this study are publicly available from the CHARLS. CHARLS data can be accessed through registration at the CHARLS website (http://charls.pku.edu.cn/). Access to the data is free for academic research purposes upon approval of the data use agreement.
