# Supplementary material for: Disability transitions and life expectancy: a national longitudinal study on social isolation and loneliness in China
Source: Glob Health Action. 2026 Apr 7;19(1):2647315. doi: 10.1080/16549716.2026.2647315 (PMC13059030; doi:10.1080/16549716.2026.2647315)
Supplement: Supplementary material_order.docx [file ZGHA_A_2647315_SM5556.docx]

**Supplementary Materials**

Disability Transitions and Life Expectancy: A National Longitudinal Study on Social Isolation and Loneliness in China

**Catalogue**

**Methods A.1** Study design and participants

**Figure A.1** Flow chart of participant inclusion and exclusion in the China Health and Retirement Longitudinal Study (CHARLS)

**Figure A.2** Life Expectancy Differences by Social Isolation Status Among Female Participants

**Figure A.3** Life Expectancy Differences by Social Isolation Status Among Male Participants

**Figure A.4** Life Expectancy Differences by Social Isolation Status Among Participants without Chronic Disease

**Figure A.5** Life Expectancy Differences by Social Isolation Status Among Participants with Chronic Disease

**Figure A.6** Life Expectancy Differences by Social Isolation Status Among Rural Participants

**Figure A.7** Life Expectancy Differences by Social Isolation Status Among Urban Participants

**Figure A.8** Life Expectancy Differences by Loneliness Status Among Female Participants

**Figure A.9** Life Expectancy Differences by Loneliness Status Among Male Participants

**Figure A.10** Life Expectancy Differences by Loneliness Status Among Participants without Chronic Disease

**Figure A. 11** Life Expectancy Differences by Loneliness Status Among Participants with Chronic Disease

**Figure A. 12** Life Expectancy Differences by Loneliness Status Among Rural Participants

**Figure A. 13** Life Expectancy Differences by Loneliness Status Among Urban Participants

**Table A.1** Summary of variable definitions in CHARLS

**Table A.2** Hazard Ratios for State Transitions by Joint Loneliness and Social Isolation Profile (Reference: Neither Isolated nor Lonely)

**Table A.3** Sensitivity Analyses (1-3) of Hazard Ratios for State Transitions Associated with Social Isolation and Loneliness

**Table A.4** Three-State Model Sensitivity Analysis: Hazard Ratios for Social Isolation and Loneliness

**Appendix Reference**

**Methods A.1: Study design and participants**

The China Health and Retirement Longitudinal Study (CHARLS) is a nationally representative longitudinal survey designed to examine health and economic adjustments to rapid population aging in China [1-3]. CHARLS was initiated between June 2011 and March 2012, encompassing 17,708 individual participants from 150 county-level units across 28 provinces [2, 4]. The study implements a sophisticated multistage stratified probability-proportional-to-size sampling design: initially, 150 county-level units were randomly selected from all county-level units excluding Tibet, stratified by geographic region, urban/rural classification, and per capita GDP [5]. Subsequently, three primary sampling units (PSUs) consisting of administrative villages in rural areas and neighborhoods in urban areas were selected within each county [4]. Following comprehensive mapping and listing operations to establish sampling frames, households with members aged 39 years or older were randomly selected, with one age-eligible individual chosen per household. When the selected person was 45 years or older, both the participant and spouse were interviewed using face-to-face computer-assisted personal interviews (CAPI). The baseline survey achieved a response rate of 80.5%, with nonresponse attributed to refusal (8.8%), inability to contact residents (8.2%), and other reasons (2.0%) [2, 6]. CHARLS employs biennial follow-up surveys with participants tracked in 2013, 2015, 2018, and 2020, incorporating proxy interviews when original respondents were unavailable due to migration or illness [7]. In cases where participant mortality was reported during subsequent follow-up waves, research teams attempted to locate knowledgeable family members to conduct exit interviews for obtaining mortality-related information [8]. The comprehensive data collection encompasses demographics, health status and functioning, physician-diagnosed chronic illnesses, lifestyle behaviors, cognitive assessment, depression screening, healthcare utilization, employment history, household economics, and family dynamics, supplemented by anthropometric measurements, physical performance tests, and biomarker collection [3]. The study was approved by the Biomedical Ethics Committee of Peking University (IRB00001052-11015). The data supporting the findings of this study were obtained from the China Health and Retirement Longitudinal Study (CHARLS). Researchers may access these datasets by submitting a data user agreement to the CHARLS team through their official website at http://charls.pku.edu.cn.

For the current analysis investigating disability transitions and life expectancy, data from Waves 1–5 of the CHARLS (2011–2020) were utilized. The initial sample included 25,428 participants aged 45 and older. We systematically excluded 260 participants due to missing disability state information at any wave, 2,393 individuals with incomplete baseline hobby and covariate data, and 1,821 participants with only single-wave disability data available. The final analytical cohort comprised 20,954 participants, among whom 2,184 were deceased, forming the basis for modeling disability state transitions and estimating life expectancy.


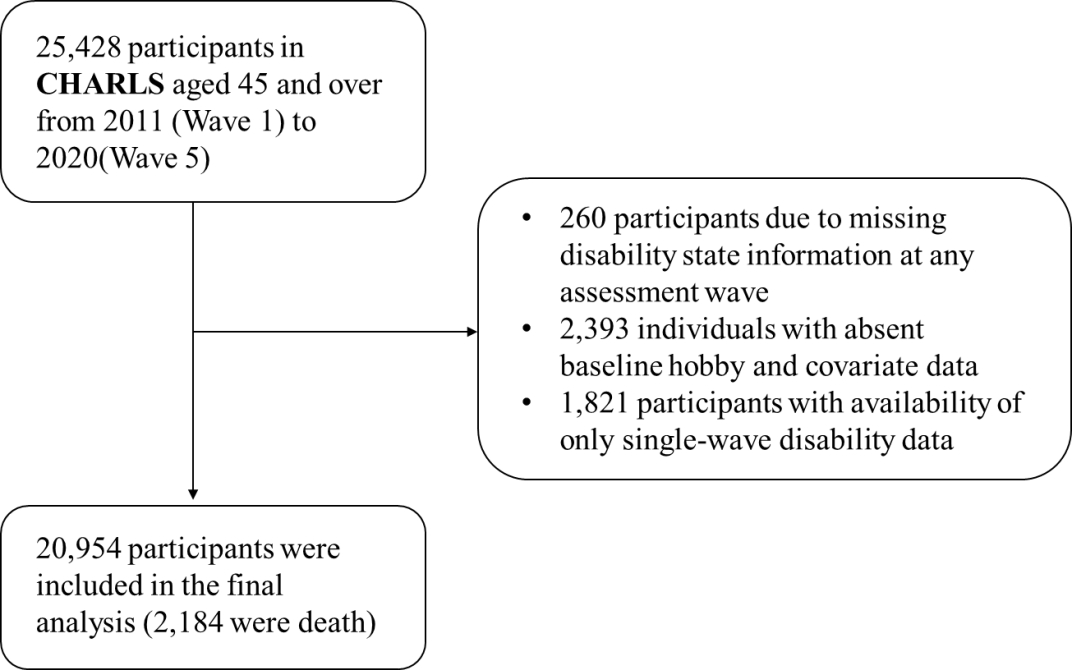


**Figure A.1** Flow chart of participant inclusion and exclusion in the China Health and Retirement Longitudinal Study (CHARLS)


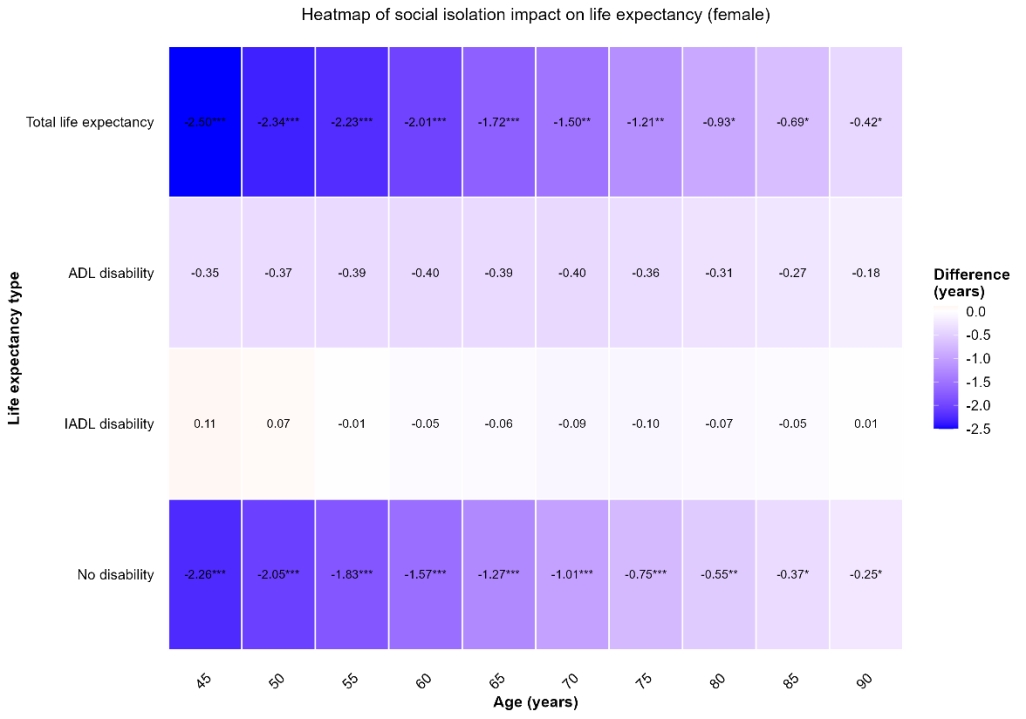


**Figure A.2** Life Expectancy Differences by Social Isolation Status Among Female Participants. Values represent differences in life expectancy (years). * indicate statistical significance levels: *** p<0.001, ** p<0.01, * p<0.05.


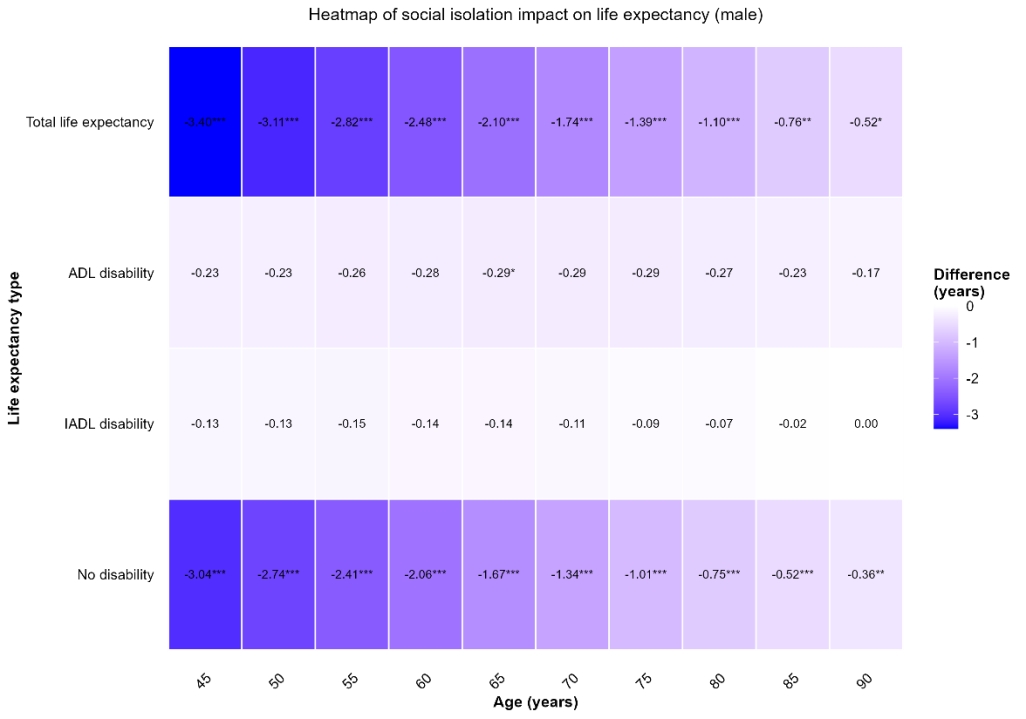


**Figure A.3** Life Expectancy Differences by Social Isolation Status Among Male Participants. Values represent differences in life expectancy (years). * indicate statistical significance levels: *** p<0.001, ** p<0.01, * p<0.05.


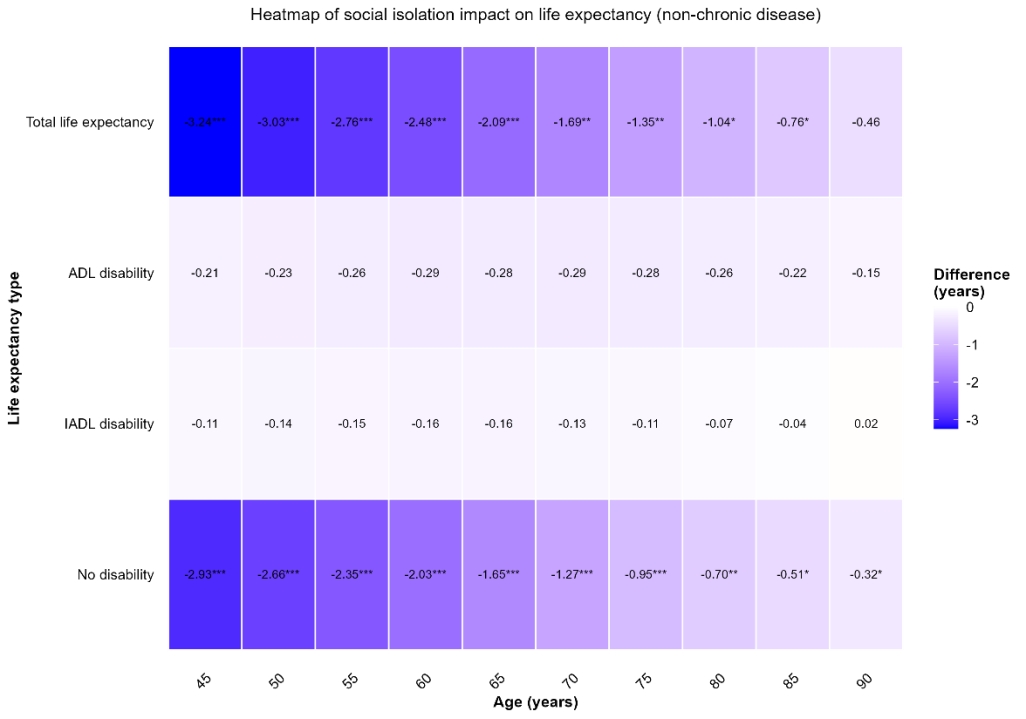


**Figure A.4** Life Expectancy Differences by Social Isolation Status Among Participants without Chronic Disease. Values represent differences in life expectancy (years). * indicate statistical significance levels: *** p<0.001, ** p<0.01, * p<0.05.


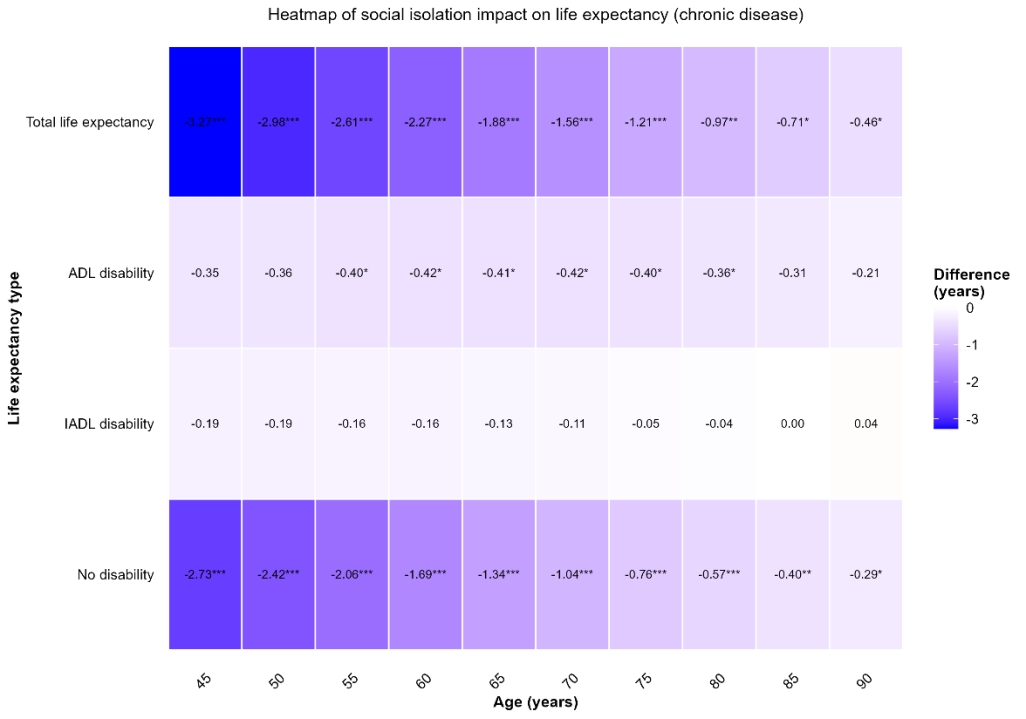


**Figure A.5** Life Expectancy Differences by Social Isolation Status Among Participants with Chronic Disease. Values represent differences in life expectancy (years). * indicate statistical significance levels: *** p<0.001, ** p<0.01, * p<0.05.


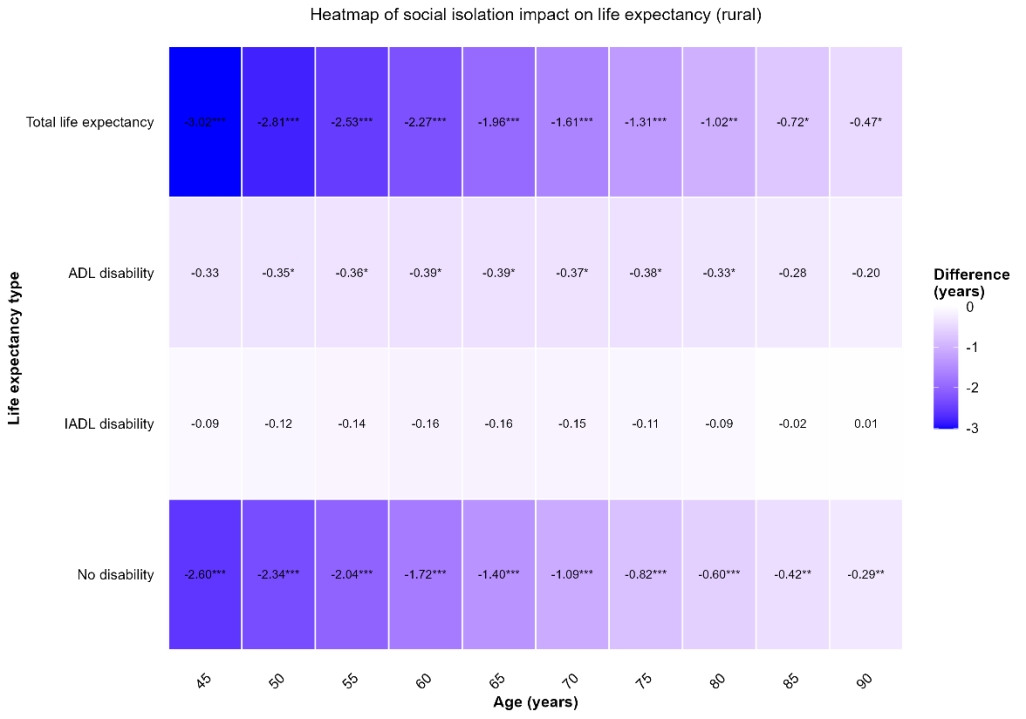


**Figure A.6** Life Expectancy Differences by Social Isolation Status Among Rural Participants. Values represent differences in life expectancy (years). * indicate statistical significance levels: *** p<0.001, ** p<0.01, * p<0.05.


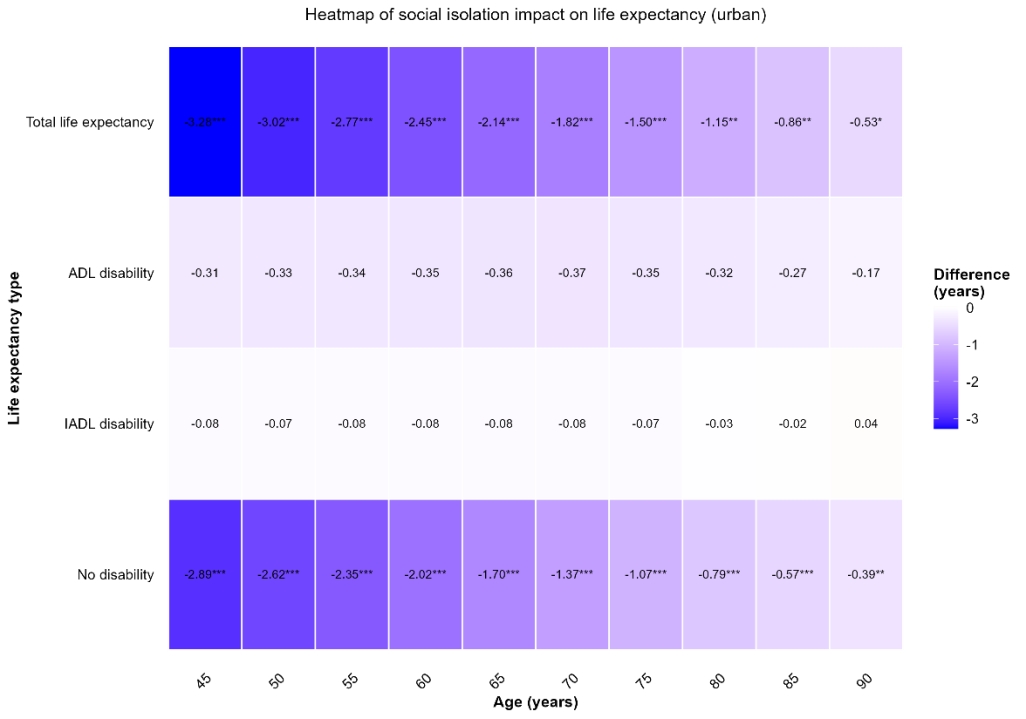


**Figure A.7** Life Expectancy Differences by Social Isolation Status Among Urban Participants. Values represent differences in life expectancy (years). * indicate statistical significance levels: *** p<0.001, ** p<0.01, * p<0.05.


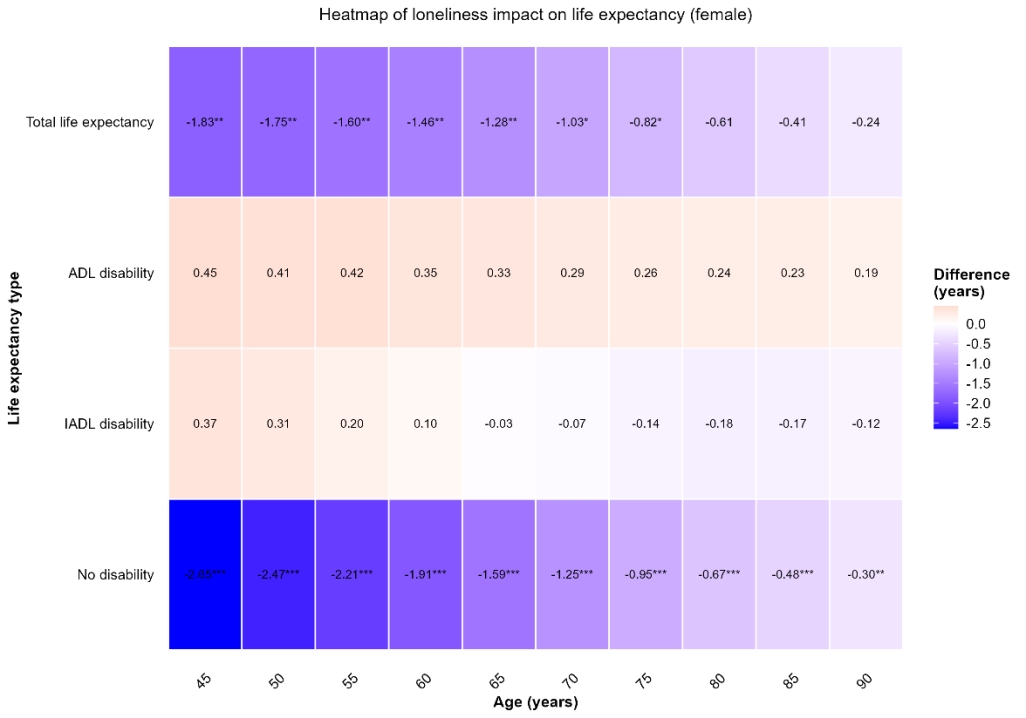


**Figure A.8** Life Expectancy Differences by Loneliness Status Among Female Participant. Values represent differences in life expectancy (years). * indicate statistical significance levels: *** p<0.001, ** p<0.01, * p<0.05.


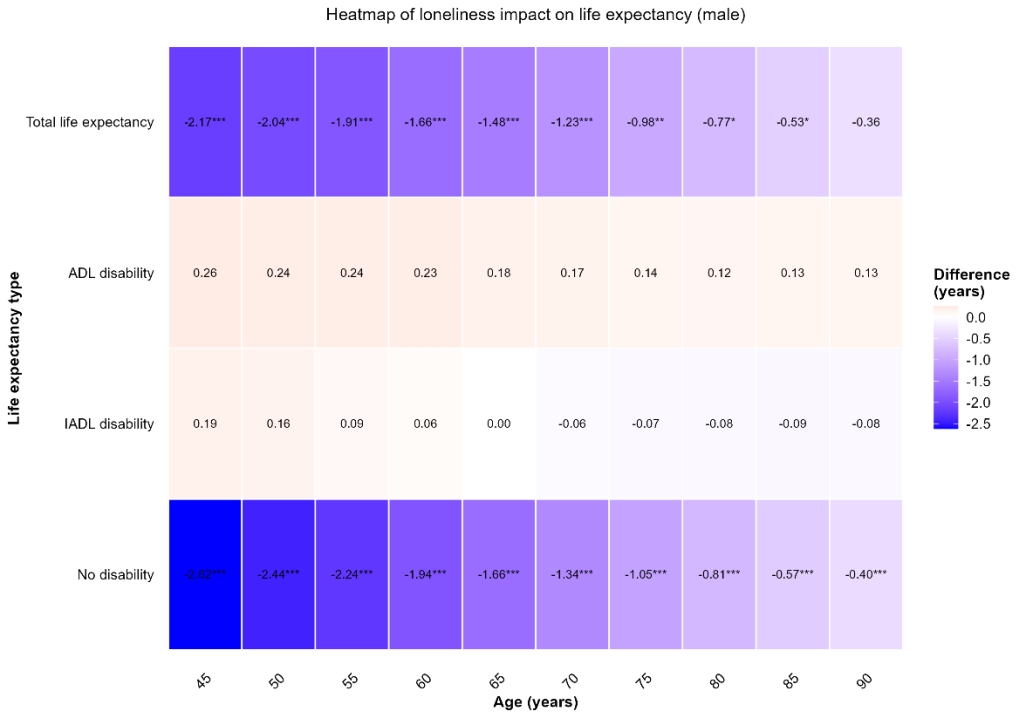


**Figure A.9** Life Expectancy Differences by Loneliness Status Among Male Participants. Values represent differences in life expectancy (years). * indicate statistical significance levels: *** p<0.001, ** p<0.01, * p<0.05.


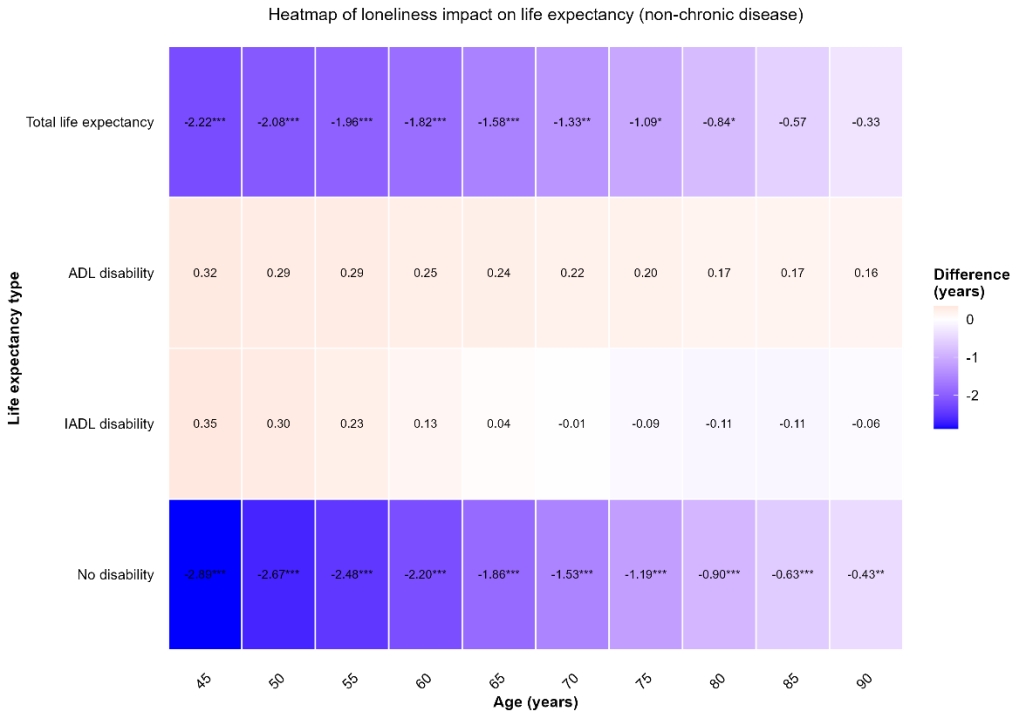


**Figure A.10** Life Expectancy Differences by Loneliness Status Among Participants without Chronic Disease. Values represent differences in life expectancy (years). * indicate statistical significance levels: *** p<0.001, ** p<0.01, * p<0.05.


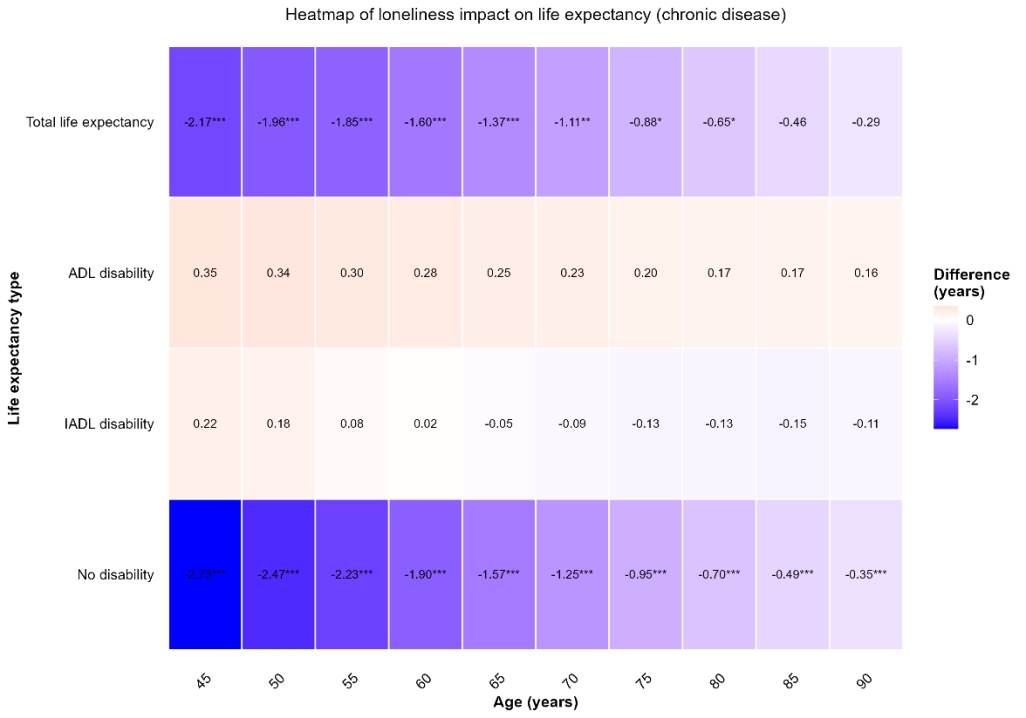


**Figure A.11** Life Expectancy Differences by Loneliness Status Among Participants with Chronic Disease. Values represent differences in life expectancy (years). * indicate statistical significance levels: *** p<0.001, ** p<0.01, * p<0.05.


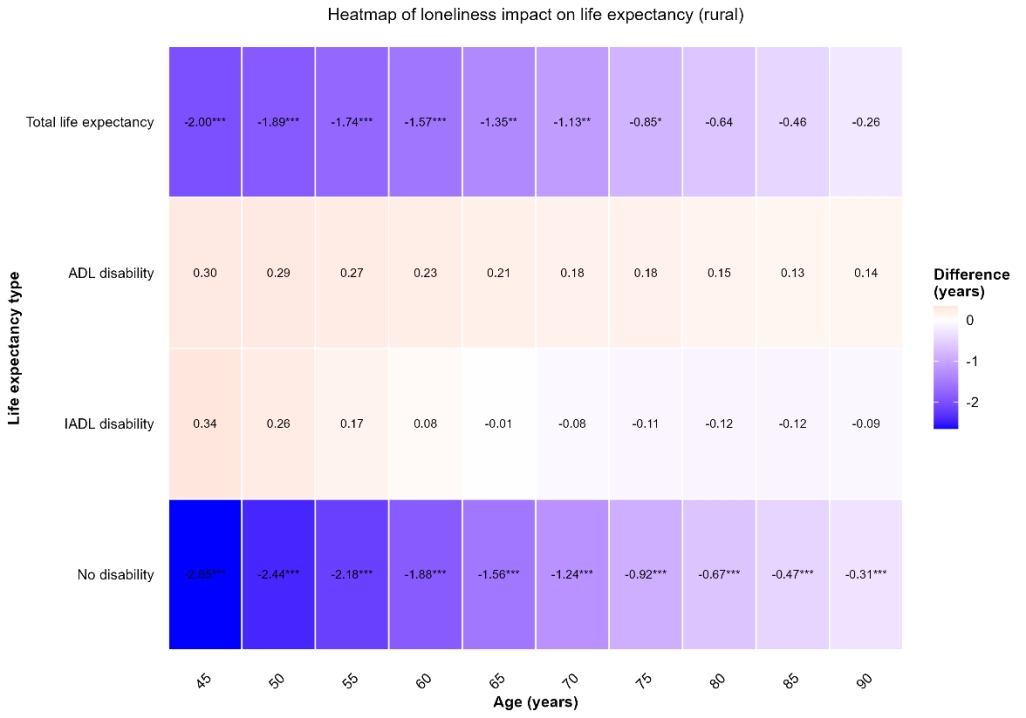


**Figure A.12** Life Expectancy Differences by Loneliness Status Among Rural Participants. Values represent differences in life expectancy (years). * indicate statistical significance levels: *** p<0.001, ** p<0.01, * p<0.05.


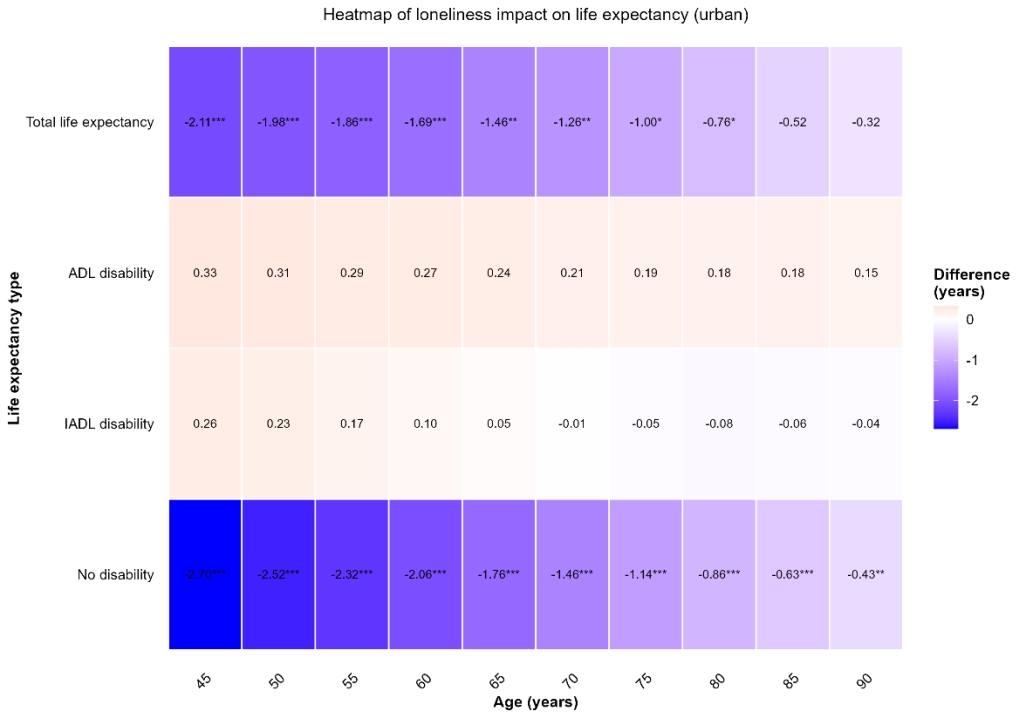


**Figure A.13** Life Expectancy Differences by Loneliness Status Among Urban Participants. Values represent differences in life expectancy (years). * indicate statistical significance levels: *** p<0.001, ** p<0.01, * p<0.05.

| **Table A.1** Summary of variable definitions in CHARLS​​ | | | |
| --- | --- | --- | --- |
| **Category** | **Variable Name** | **Variable Type** | **Detailed Description / Operationalization** |
| Primary Exposures​​ | Social Isolation​​ | Categorical (Dichotomous) ​​ | Components: (1) Marital status ("No" = married; "Yes" = unmarried), (2) Living alone (yes/no), (3) Less than weekly contact with children (yes/no), (4) Participate in social activities ("No" = participates; "Yes" = does not participate) [9, 10]. |
|  |  |  | Scoring: 1 point for each: being unmarried, living alone, contact with children < weekly, no social activity participation in the past month. Score range: 0-4. |
|  |  |  | Analysis Cut-off: Non-isolated (score < 2) vs. Socially isolated (score ≥ 2) [11]. |
|  | Loneliness​​ | Categorical (Dichotomous) | Measure: Single item: "How often did you feel lonely during the past week?" |
|  |  |  | Original Scale: 4-point Likert: rarely/never (<1 day), some/a little time (1-2 days), occasionally/moderate time (3-4 days), most/all the time (5-7 days). |
|  |  |  | Analysis Cut-off: Not lonely (rarely/never) vs. Lonely (some of the time or more frequently) [9, 10]. |
| Outcome Variables​​ | Functional Disability (ADL/IADL)​​ | Dichotomous​​ | Measures [12]: |
|  |  |  | 1. Activities of Daily Living (ADL): 6 basic tasks (dressing, bathing, eating, etc.). |
|  |  |  | 2. Instrumental ADL (IADL): 6 complex tasks (preparing meals, shopping, managing finances, etc.). |
|  |  |  | Definition: Disability defined as having difficulty or requiring assistance with at least one activity in ADL or IADL [10]. |
|  | Disability State (for Multi-State Analysis)​​ | Multinomial​​ | Four mutually exclusive states [13]: |
|  |  |  | 1. No disability: No ADL or IADL limitations. |
|  |  |  | 2. Mild disability only: ≥1 IADL limitation, but no ADL limitations. |
|  |  |  | 3. Severe disability: ≥1 ADL limitation (with or without IADL limitations). |
|  |  |  | 4. Death: Absorbing state. |
| Covariates​​ | Sociodemographic Factors​​ | Categorical | Includes: Gender (Male, Female), Age group (45–54, 55–64, 65–74, ≥75), Educational attainment (Less than secondary school, Secondary or above), Residence (Urban/Rural) |
|  | Behavioral & Lifestyle Factors​​ | Categorical​​ | Includes: Smoke (No, Yes), Drink (No, Yes). |
|  | Health-Related Factors​​ | Categorical​​ | Includes: Chronic diseases (Defined as the presence of at least one physician-diagnosed condition from the following: hypertension, diabetes, heart disease, stroke, chronic lung disease, or cancer. [No, Yes]);  CESD-9: Assessed using a 9-item version derived from the 10-item Center for Epidemiological Studies Depression Scale (CESD-10) by excluding the loneliness item. The total score ranges from 0 to 27.  Scoring: Seven negative items (e.g., "I was bothered by things," "I felt depressed") and two positive items ("I felt hopeful about the future," "I was happy") were rated on a 4-point Likert scale based on frequency in the past week: 1="Rarely or none of the time" (<1 day), 2="Some or a little of the time" (1-2 days), 3="Occasionally or a moderate amount of the time" (3-4 days), 4="Most or all of the time" (5-7 days).  For the seven negative items, the response score was used directly (1→0, 2→1, 3→2, 4→3). For the two positive items, the score was reversed (1→3, 2→2, 3→1, 4→0).  Depressive symptoms: No (<9), Yes (≥9) [14] |
|  |  |  |  |

**Table A.2** Hazard Ratios for State Transitions by Joint Loneliness and Social Isolation Profile (Reference: Neither Isolated nor Lonely)

|  | Isolated Only |  | Lonely Only |  | Both |
| --- | --- | --- | --- | --- | --- |
|  | HR(95%CI) |  | HR(95%CI) |  | HR(95%CI) |
| State 1 - State 2 | 1.19 (0.97, 1.46) |  | 1.22** (1.06, 1.40) |  | 1.32* (1.05, 1.66) |
| State 1 - State 3 | 1.22 (0.96, 1.54) |  | 1.39*** (1.23, 1.57) |  | 1.22 (0.94, 1.59) |
| State 2 - State 1 | 0.77* (0.61, 0.98) |  | 0.95 (0.83, 1.08) |  | 0.80 (0.61, 1.05) |
| State 2 - State 3 | 1.19 (0.94, 1.50) |  | 1.03 (0.86, 1.22) |  | 1.40** (1.10, 1.77) |
| State 2 - State 4 | 1.62 (0.40, 6.59) |  | 1.04 (0.41, 2.61) |  | 1.08 (0.20, 5.82) |
| State 3 - State 1 | 1.38** (1.10, 1.74) |  | 0.88 (0.75, 1.04) |  | 0.94 (0.72, 1.24) |
| State 3 - State 2 | 1.01 (0.72, 1.42) |  | 0.99 (0.81, 1.22) |  | 0.98 (0.71, 1.37) |
| State 3 - State 4 | 1.26** (1.07, 1.47) |  | 0.97 (0.85, 1.10) |  | 1.21* (1.04, 1.41) |

Note: HR: Hazard ratio; CI: Confidence Interval. State 1 = no disability; State 2 = mild disability; State 3 = severe disability; State 4 = death; * indicates 5% significant ratio. *p<0.05, **p<0.01, ***p<0.001. This model was adjusted for age, gender, residence, education level, chronic diseases, healthy behaviors (drink and smoke) and depression.

**Table A.3** Sensitivity Analyses (1-3) of Hazard Ratios for State Transitions Associated with Social Isolation and Loneliness

| State Transition | Analysis 1 | |  | Analysis 2 | |  | Analysis 3 | |
| --- | --- | --- | --- | --- | --- | --- | --- | --- |
|  | Social Isolation | Loneliness |  | Social Isolation | Loneliness |  | Social Isolation | Loneliness |
|  | HR(95%CI) | HR(95%CI) |  | HR(95%CI) | HR(95%CI) |  | HR(95%CI) | HR(95%CI) |
| State 1 - State 2 | 1.20** (1.07,1.34) | 1.23*** (1.12,1.36) |  | 1.26* (1.05,1.52) | 1.42* (1.23,1.64) |  | 1.15* (1.01,1.31) | 1.15* (1.03,1.29) |
| State 1 - State 3 | 1.24 (0.90,1.70) | 0.96 (0.72,1.29) |  | 0.97 (0.73,1.30) | 1.03 (0.79,1.34) |  | 1.46 (0.97,2.19) | 1.00 (0.67,1.48) |
| State 2 - State 1 | 0.89 (0.79,1.01) | 0.89* (0.81,0.98) |  | 1.14 (0.92,1.42) | 1.19* (1.00,1.41) |  | 0.97 (0.82,1.13) | 0.87* (0.77,1.00) |
| State 2 - State 3 | 1.04 (0.88,1.22) | 1.15 (0.98,1.35) |  | 1.32 (0.96,1.81) | 1.25 (0.96,1.64) |  | 1.06 (0.84,1.34) | 0.93 (0.74,1.17) |
| State 2 - State 4 | 0.90 (0.52,1.54) | 1.11 (0.77,1.59) |  | 1.06 (0.64,1.74) | 0.98 (0.68,1.43) |  | 1.14 (0.84,1.55) | 1.07 (0.84,1.35) |
| State 3 - State 1 | 1.15 (0.73,1.82) | 0.69 (0.45,1.07) |  | 0.34 (0.05,2.19) | 0.55 (0.30,1.02) |  | 0.71 (0.34,1.48) | 0.65 (0.41,1.01) |
| State 3 - State 2 | 0.83 (0.65,1.05) | 1.06 (0.88,1.29) |  | 1.48 (0.88,2.48) | 1.15 (0.74,1.8) |  | 1.15 (0.78,1.71) | 0.86 (0.64,1.16) |
| State 3 - State 4 | 1.35*** (1.17,1.56) | 0.93 (0.81,1.07) |  | 1.30* (1.11,1.51) | 0.90 (0.77,1.05) |  | 1.37*** (1.14,1.65) | 0.84 (0.71,1.01) |

Note: Analysis 1: Age ≥50 years. Analysis 2: ADL-only disability definition. Analysis 3: Age ≥50 years with wave-complete data (time-varying complete cases). State 1 = no disability; State 2 = mild disability; State 3 = severe disability; State 4 = death. HR = Hazard Ratio; CI = Confidence Interval. *p<0.05, **p<0.01, ***p<0.001.

**Table A.4** Three-State Model Sensitivity Analysis: Hazard Ratios for Social Isolation and Loneliness

| State Transition | Social isolation | Loneliness |
| --- | --- | --- |
|  | HR(95%CI) | HR(95%CI) |
| State 1 - State 2 | 1.19*** (1.1,1.29) | 1.16*** (1.09,1.24) |
| State 1 - State 3 | 2.83 (0.82,9.73) | 1.10 (0.39,3.07) |
| State 2 - State 1 | 0.94 (0.86,1.04) | 0.84*** (0.78,0.91) |
| State 2 - State 3 | 1.21*** (1.09,1.35) | 1.02 (0.92,1.12) |

Note: State 1 = no disability; State 2 = disability; State 3 = death. HR = Hazard Ratio; CI = Confidence Interval. *p<0.05, **p<0.01, ***p<0.001.

**References**

1. Chen Y, Zhang S, Wang Y, Li H, Liu M, Xu Y: **Association of TyG index and central obesity with hypertension in middle-aged and elderly Chinese adults: a prospective cohort study**. *Scientific Reports* 2024, **14**(1):2235.

2. Zhao Y, Hu Y, Smith JP, Strauss J, Yang G: **China Health and Retirement Longitudinal Study (CHARLS) – 2011-2012 National Baseline Users’ Guide**. Beijing: National School of Development, Peking University; 2013.

3. Chen X, Wang C, Zhang Y: **China Health and Retirement Longitudinal Study (CHARLS)**. In: *Encyclopedia of Gerontology and Population Aging.* edn. Edited by Gu D, Dupre ME. Cham: Springer International Publishing; 2019: 1-9.

4. Gao Q, Zhou Y, Wang L, Li J, Zhang T, Liu X: **Multistate survival modelling of multimorbidity and transitions across health needs states and death in an ageing population**. *Journal of Epidemiology and Community Health* 2024, **78**(4):212-219.

5. Qin VM, Zhang L, Wang P, Liu Y, Chen X, Li H: **Temporal trends and variation in out-of-pocket expenditures and patient cost sharing: evidence from a Chinese national survey 2011–2015**. *International Journal for Equity in Health* 2021, **20**(1):143.

6. Feng Z, Wang W, Li J, Liu M, Zhang Y, Xu H: **The relationship between depressive symptoms and activity of daily living disability among the elderly: results from the China Health and Retirement Longitudinal Study (CHARLS)**. *Public Health* 2021, **198**:75-81.

7. Zhao Y, Hu Y, Smith JP, Strauss J, Yang G: **Cohort profile: the China Health and Retirement Longitudinal Study (CHARLS)**. *Int J Epidemiol* 2014, **43**(1):61-68.

8. Lei X, Strauss J, Tian M, Zhao Y: **Living arrangements of the elderly in China: evidence from the CHARLS national baseline**. *China Economic J* 2015, **8**(3):191-214.

9. Song Y, Wang A, Li J, Chen X, Liu Y, Zhang M: **Social isolation, loneliness, and incident type 2 diabetes mellitus: results from two large prospective cohorts in Europe and East Asia and Mendelian randomization**. *eClinicalMedicine* 2023, **64**:102236.

10. Guo L, Zhang Y, Wang C, Li H, Liu X, Xu Y: **Social isolation, loneliness and functional disability in Chinese older women and men: a longitudinal study**. *Age and Ageing* 2021, **50**(4):1222-1228.

11. Lin L, Wang W, Li J, Liu M, Zhang Y, Xu H: **Association of Adverse Childhood Experiences and Social Isolation With Later-Life Cognitive Function Among Adults in China**. *JAMA Network Open* 2022, **5**(11):e2241714.

12. Gill TM, Hardy SE, Williams CS: **Underestimation of disability in community-living older persons**. *Journal of the American Geriatrics Society* 2002, **50**(9):1492-1497.

13. Fong JH, Feng J: **Comparing the loss of functional independence of older adults in the U.S. and China**. *Archives of Gerontology and Geriatrics* 2018, **74**:123-127.

14. Liu X, Wang Y, Li J, Zhang T, Zhou Y, Chen S: **Social isolation and depressive symptoms among older adults with different functional status in China: A latent class analysis**. *Journal of Affective Disorders* 2025, **375**:478-485.
